# Prevalence, antibiogram and molecular characterization of *Listeria monocytogenes* from ruminants and humans in New Valley and Beheira Governorates, Egypt

**DOI:** 10.1186/s12917-024-04138-0

**Published:** 2024-07-06

**Authors:** Sotohy A. Sotohy, Yasser F. Elnaker, Aya M. Omar, Nehal K. Alm Eldin, Mohamed Said Diab

**Affiliations:** 1 Department of Animal, Poultry and Environmental Hygiene, Faculty of Veterinary Medicine, Assiut University, Asyut, 71515 Egypt; 2 Department of Infectious Diseases, Faculty of Veterinary Medicine, New Valley University, El-Kharga, 1062001 New Valley Egypt; 3 Department of Animal Hygiene and Zoonoses, Faculty of Veterinary Medicine, New Valley University, Kharga Oasis, 1062001 New Valley Egypt

**Keywords:** *L. monocytogenes*, Cattle, Goats, Sheep, *16S rRNA* gene, *InlB* genes

## Abstract

**Background:**

Listeriosis is a global health threat to both animals and humans, especially in developing countries. This study was designed to isolate *Listeria monocytogenes* from faeces; environmental samples; and cow, sheep and goat milk, as well as human stool, to study its molecular characteristics and antibiotic sensitivity in the New Valley and Beheira Governorates, Egypt. The isolation and identification of *L. monocytogenes* were carried out using traditional culture and biochemical methods, followed by antibiography, genus confirmation of some isolates and detection and sequencing of *InlB* genes via PCR.

**Results:**

Out of 2097 examined samples, the prevalence of *L. monocytogenes* was 13.4% in animals; the prevalence was 9.2%, 2.4%, 25.4%, 4%, 42.4%, and 6.4% in cattle faeces, cattle milk, sheep faeces, sheep milk, goat faeces, and goat milk, respectively. However, the prevalence of *L. monocytogenes* was 8.3% in human samples. Both animal and human isolates showed 100% resistance to trimethoprim-sulfamethoxazole, and the isolates showed the highest sensitivity to flumequine (100%), amikacin (99.2%), gentamicin (97.6%), and levofloxacin (94.6%). Multidrug resistance (MDR) was detected in 86.9% of the tested isolates. The *16 S rRNA* and *inlB* genes were detected in 100% of the randomly selected *L. monocytogenes* isolates. Phylogenetic analysis of three isolates based on the *inlB* gene showed 100% identity between faecal, milk and human stool isolates.

**Conclusions:**

Faeces and milk are major sources of listeriosis, and the high degree of genetic similarity between animal and human isolates suggests the possibility of zoonotic circulation. The high prevalence of MDR *L. monocytogenes* in both animal and human samples could negatively impact the success of prevention and treatments for animal and human diseases, thereby imposing serious risks to public health.

**Supplementary Information:**

The online version contains supplementary material available at 10.1186/s12917-024-04138-0.

## Background

Listeriosis is a significant food-borne zoonosis with severe clinical consequences. *L monocytogenes*, which belongs to the genus *Listeria*, is a rod-shaped, gram-positive bacterium that is motile at 10 °C to 25 °C, non-spore-forming and facultatively anaerobic; it is extensively distributed in many natural environments, such as the water, debris, and soil. This disease can affect a wide range of domestic animals, birds, wild animals, and fish in sporadic cases or outbreaks. Domestic animals are infected mainly by the ingestion of contaminated water and feed. Moreover, animals can be infected by inhalation or venereal transmission, and infected animals shed these bacteria in faeces, milk, urine, uterine discharge, or nasal discharge [1–3].

Listeriosis in farm animals is a major problem that increases the risk of transmission to humans. The major sources of human listeriosis are animal products, which are contaminated with animal faeces; *L. monocytogenes* can be transmitted by direct or indirect contact with infected animals [4, 5]. *L. monocytogenes* can be shed in the faeces of diseased, recovered, or asymptomatic animals, causing contamination of the environment, thereby increasing intraherd transmission, accidental spread to other herds, and increasing the risk of human infection [6, 7].

In humans, Listeriosis is an international public health concern because it has the greatest fatality rate among food-borne pathogens, with fatality rates of up to 20–30% [8]. The most important sources of human listeriosis are the consumption of contaminated raw or undercooked food such as milk, soft cheese, unpasteurized dairy products, raw vegetables, meat products, and poultry [9]. Moreover, listeriosis causes watery diarrhoea, fever, abdominal pain, and vomiting in noninvasive patients; usually persists for approximately one to three days; and is self-limiting [10]. However, listeriosis causes severe invasive disease and serious clinical signs in children, immunocompromised patients and elderly individuals, including meningitis, endocardia, septicemia, conjunctivitis, and flu-like symptoms [11, 12].

Polymerase chain reaction (PCR) is an effective procedure for the bacteriological examination of *Listeria* species and for evaluating their pathogenicity. The *16 S rRNA* gene is used for the molecular identification and differentiation of *L. monocytogenes.* The pathogenicity and severity of *L. monocytogenes* depend mainly on the presence or absence of virulence genes. The internalin B (*inlB)* gene is a common genetic marker for identifying the pathogenicity of *L. monocytogenes*, as it plays the most important role in the adhesion, invasion and production of a biofilm that allows for bacterial penetration of host cells [13, 14].

Antibiotics are very important for the treatment of listeriosis, but unfortunately, the effectiveness of antibiotics decreases as the antimicrobial resistance of *Listeria* increases. The random use and misuse of antibiotics in communities and animal farms results in increased *L. monocytogenes* resistance to antibiotics and the development of a critical level of MDR. This resistance to antibiotics poses a real threat to human and animal health, as the number of deaths related to this problem is estimated to be more than 700 thousand annually [15, 16].

The aim of the present study was to identify and confirm *L. monocytogenes* strains recovered from the milk and faeces of cows, sheep and goats, as well as human stool. Moreover, the prevalence and antibiotic resistance and genetic similarity of the examined isolates were determined.

## Results

A total of 262 *L. monocytogenes* isolates were recovered from 2097 examined samples from different sources, including fresh milk, animal faeces, and human stool. The results in Table (1) show that the overall prevalence of *L. monocytogenes* was 13.4% and 8.3% in animals and humans, respectively. The prevalence of *L. monocytogenes* was 31%, 2.4%, 47.1%, 4.04%, 55.8%, and 6.4% in cattle faeces, cattle milk, sheep faeces, sheep milk, goat faeces, and goat milk, respectively, as shown in Table (2).

**Table 1:** Overall prevalence of *L. monocytogenes* in animal and human samples

Sample	Total No.	Positive No.	%	Chi-square	Asymp. Sig.
Animal	1703	229	13.45	1.19	0.275
Human	394	33	8.38		
**Total**	**2097**	**262**	**10.9**		

**Table 2:** Prevalence of *L. monocytogenes* in different animal’s samples

Animal	Type of samples	Total No.	Positive No.	%	Chi-square	Asymp. Sig.
Cattle	Feces	541	50	9.24	4.455	0.035*
	Milk	414	10	2.42		
Sheep	Feces	319	81	25.39	15.207	0.000***
	Milk	173	7	4.05		
Goat	Feces	179	76	42.46	27.000	0.000***
	Milk	77	5	6.49		
**Total**		**1703**	**229**	**15**		

**Fig. 1:**
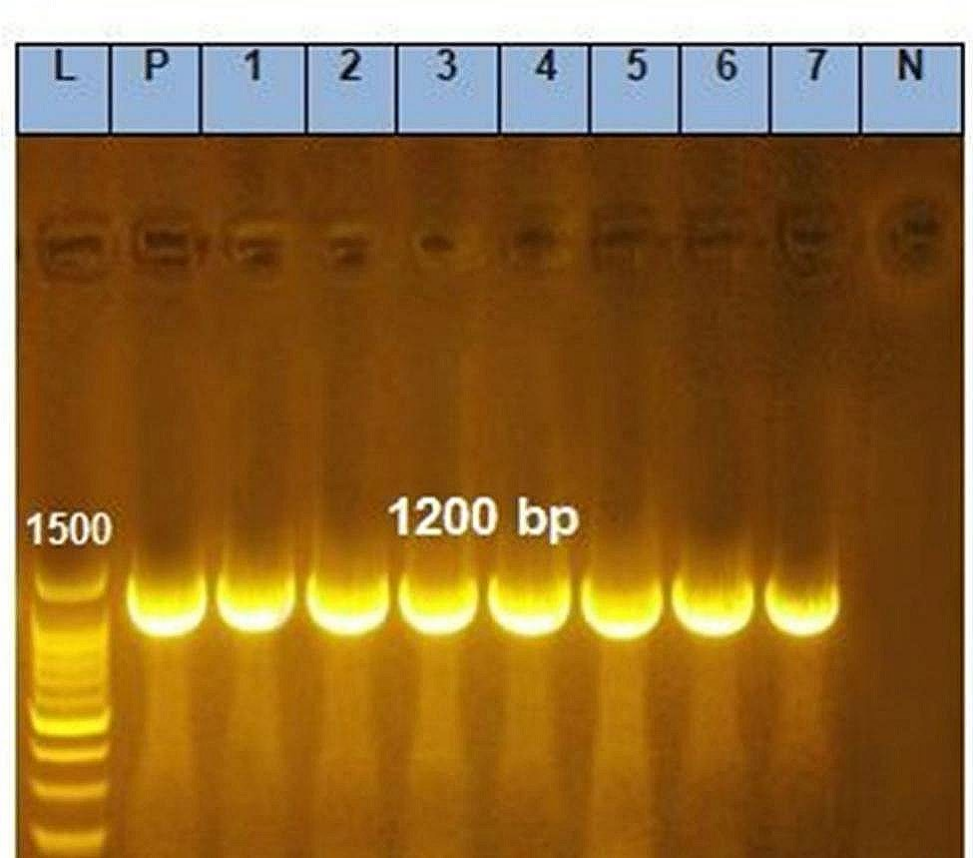
Agarose gel electrophoresis of amplified *16s rRNA* gene PCR product (1200 bp)

**Fig. 2:**
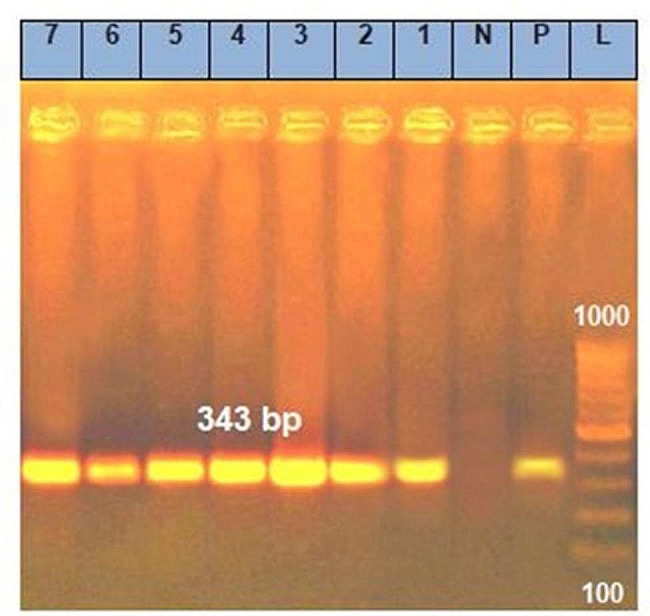
Agarose gel electrophoresis of amplified *inlB* gene PCR product (343 bp)

Seven randomly selected isolates were subjected to PCR to evaluate the presence of the *16 S rRNA* and *inlB* virulence genes. The prevalence of the *16 S rRNA* and *inlB* genes was 100%, as shown in Table (3) and Figs. 1 and 2.

**Table 3:** Occurrence of virulence genes in some *L. monocytogenes* isolates

Tyoe of examined samples	No. of L. monocytogenes isolates	16 S rRNA		inlB	
		+ve No.	+ve %	+ve No.	+ve %
Cattle feces	1	1	100	1	100
Cattle milk	1	1	100	1	100
Sheep feces	1	1	100	1	100
Sheep milk	1	1	100	1	100
Goat feces	1	1	100	1	100
Goat milk	1	1	100	1	100
Human stool	1	1	100	1	100
Total	7	7	100	7	100

Overall, 130 *L. monocytogenes* isolates were tested for antibiotic susceptibility to 12 of the most commonly used antibiotics in the study area. Both animal and human isolates showed 100% resistance to trimethoprim-sulfamethoxazole. In contrast, the isolates showed the highest sensitivity to the following antibiotics: 100% sensitivity to flumequine, 99.2% sensitivity to amikacin, 97.6% sensitivity to gentamicin, and 94.6% sensitivity to levofloxacin, as shown in Table 4. The data in Table (5) show that the mean MAR indices of *L. monocytogenes isolated* from animal and human samples were 0.435 and 0.375, respectively.

**Table 4:** Antimicrobial resistance of isolated *L. monocytogenes* from animal and human samples

Antimicrobial agents	Sensitive		Intermediate		Resistant	
	No.	%	No.	%	No.	%
Amikacin (AK)	126	99.23	0	0	1	0.77
Amoxicillin	78	60	15	11.54	37	28.46
Ampicillin	93	71.54	15	11.54	22	16.92
Apramycin (APR)	54	41.5	7	5.38	69	53.08
Flumequine	130	100	0	0	0	0
Gentamicin (CN)	126	97.69	3	2.31	0	0
Levofloxacin	123	94.62	5	3.85	15	1.15
Lincomycin (MY)	23	17.7	4	3.08	103	79.23
Norfloxacin (NOR)	95	73.31	13	10	22	16.93
Rifampicin (RD)	23	29.9	16	12.30	91	70
Trimethoprime-sulfamethoxazole (SXT)	0	0	0	0	130	100

**Table 5:** Mean value of MAR index of from animal and human samples

Source of isolates	Mean of MAR index	Mean
Cattle samples	0.343	0.435
Sheep samples	0.4	
Goat samples	0.463	
Human samples	0.375	0.375

**Fig. 3:**
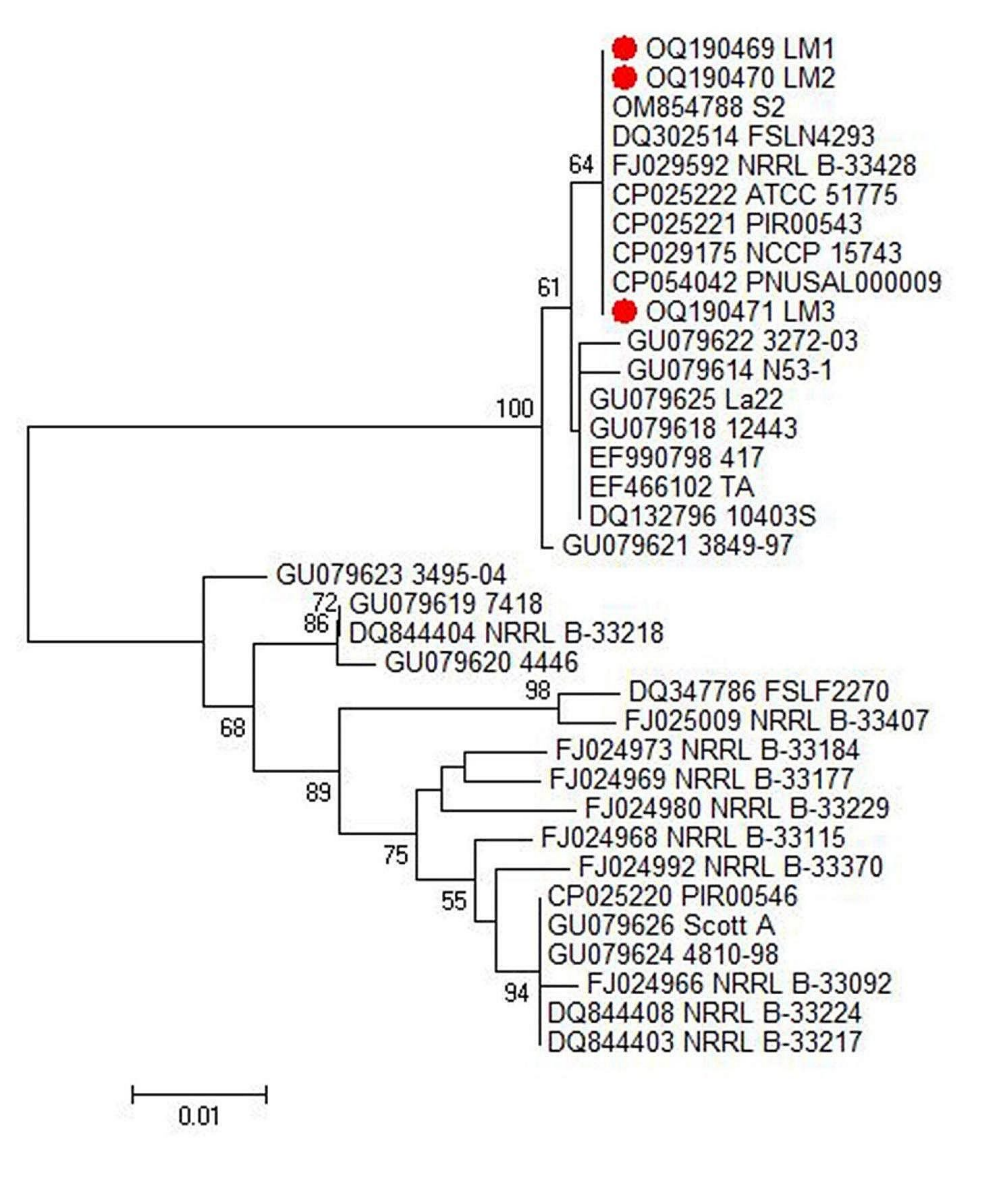
Phylogenetic tree of human and animal *L. monocytogenes* isolates based on *inlB* gene

**Fig. 4:**
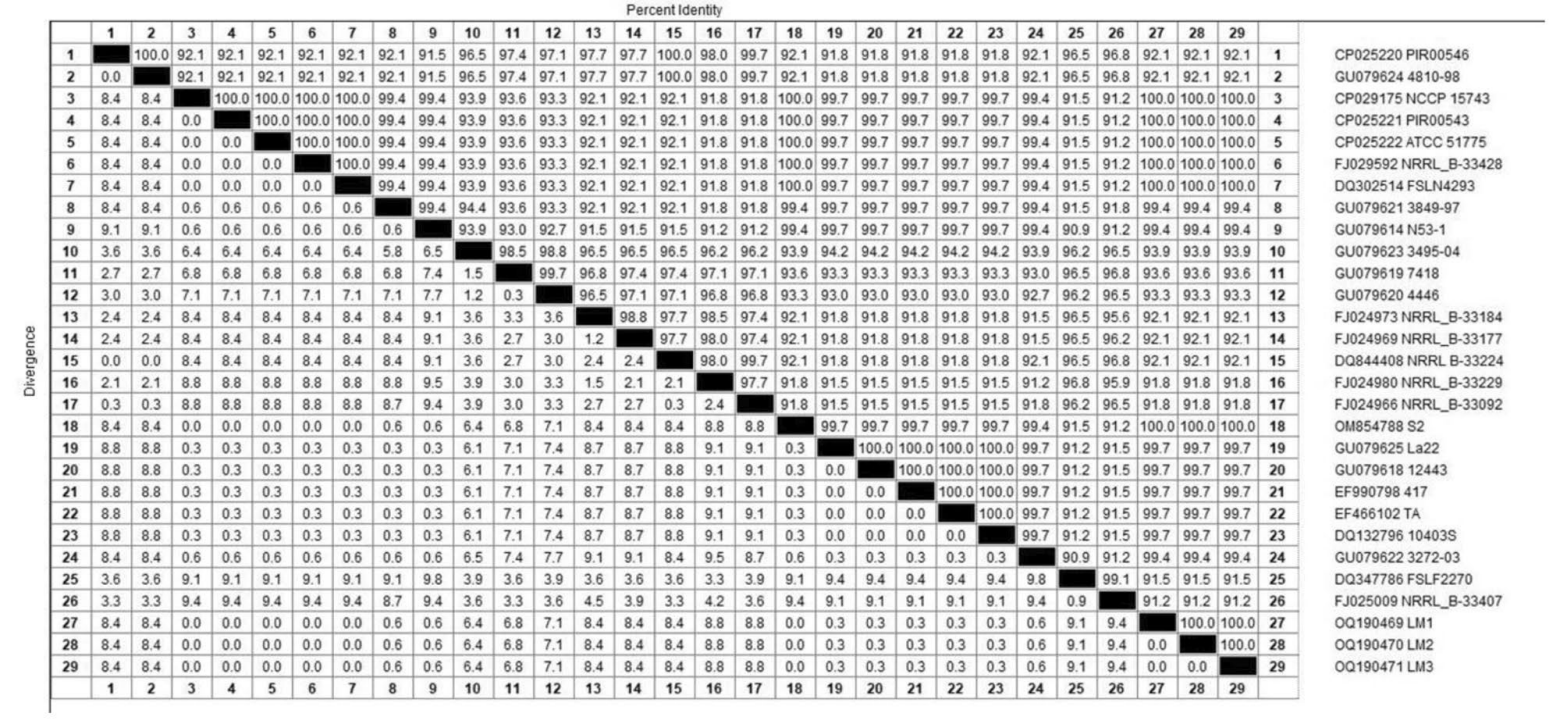
Phylogenetic analysis of human and animal *L. monocytogenes* isolates based on *inlB* gene sequencing

As shown in Figs. 3 and 4, the DNA sequences of the *inlB* genes, which represent faecal, milk and stool isolates, exhibited 100% identity with many other isolates in GenBank. The accession numbers of the analysed isolates have been deposited in GenBank as follows: faeces samples (accession number OQ190470), milk samples (accession number OQ190469) and human stool samples (accession number OQ190471). The phylogenetic tree revealed two main clusters based on *inlB* gene sequencing. The first cluster, which includes the isolates of this study, has high genetic similarity between the isolates. The second cluster contains many subclusters.

## Discussion

*Listeriosis* is one of the most severe food-borne diseases and poses a great risk to public health because it is the most fatal food-borne disease worldwide [17].

Our results showed that the overall prevalence of *L. monocytogenes* was 13.4% in animals, and this result was confirmed by another Egyptian study [18]. In contrast, other studies reported different prevalences (35.5% and 10.4%) of *L. monocytogenes* in animals [19, 20]. However, the prevalence of *L. monocytogenes* in human samples was 8.3%, which is lower than that reported in some previous studies (12.5% and 10%) [21, 22]. Other authors were unable to isolate *L. monocytogenes* from human samples [23, 24]. Our results revealed that the occurrence of *L. monocytogenes* was greater in animals than in humans, but there was no significant difference between the two groups (P value = 0.275).

The prevalence of *L. monocytogenes* was 9.2%, 2.4%, 25.4%, 4.04%, 42.4%, and 6.4% in cattle faeces, cattle milk, sheep faeces, sheep milk, goat faeces, and goat milk, respectively. A nearly similar prevalence in cattle faeces, cattle milk, sheep milk, and goat milk was reported in previous studies [25–27]. However, lower prevalence rates in goat faeces (23.3%) and sheep faeces (8%) were reported in other studies [28, 29]. These results indicated that *L. monocytogenes* was more abundant in faeces than in milk samples. The greater prevalence of *L. monocytogenes* in faeces than in milk can be attributed to a combination of seasonal effects, farm management practices, animal health and environmental conditions that favour the survival and proliferation of this pathogen in faecal material more than in the environment where milk is produced [20, 30, 31]. Therefore, animal faeces are considered the most potent source of transmission of *L. monocytogenes* to humans, other animals and milk [32]; thus, faeces from infected animals serve as the main source of *L. monocytogenes* environmental contamination [33].

This variation in the prevalence rates of *L. monocytogenes* in other studies might be due to variations in season, geographical location, animal farming practice, sample type, and isolation technique [34].

PCR is an effective method for the molecular confirmation of isolated *L. monocytogenes* via detection of the *16 S rRNA* gene and for studying its pathogenicity via detection of the *inlB* gene [15]. Our results confirmed that the *16 S rRNA* and *inlB* genes were detected in 100% of the seven randomly selected *L. monocytogenes* isolates, and these results were previously reported in other studies [35, 36]; however, in another study, *inlB* genes were detected in only 40% of the examined samples [25]. The differences in the distribution of these genes may be due to the different sample sources, *L. monocytogenes* serotypes, or mutations in these genes [2, 3].

Listeriosis is one of the most common causes of acute gastroenteritis in humans, and humans are usually treated with antibiotics to overcome infection [10]. Unfortunately, the overuse of antibiotics in animal and human medicine has resulted in the development of antimicrobial-resistant bacteria, which have become a serious problem worldwide.

Antimicrobial resistance refers to the ability of a microorganism to survive and reproduce in the presence of previously effective antibiotic doses [35]. The isolated *L. monocytogenes* strains showed the highest resistance to trimethoprim-sulfamethoxazole (100%), followed by lincomycin (79.2%) and rifampicin (70%). These results are similar to those reported in other studies [18, 37, 38]. Our results disagree with the results of other studies that reported that trimethoprim-sulfamethoxazole, rifampicin, and lincomycin were effective against *L. monocytogenes* [36, 39, 40].

The highest sensitivity levels in this study were reported for flumequine (100%), followed by amikacin (99.2%), gentamicin (97.6%), levofloxacin (94.6%), norfloxacin (73.1%), ampicillin (71.5%), and amoxicillin (60%). These results are consistent with those of other authors who reported high sensitivity rates to levofloxacin, ampicillin, and norfloxacin [35, 38, 41]. In contrast, our results disagree with the results of other authors who found that *L. monocytogenes* isolates were resistant to gentamicin, amikacin, norfloxacin, amoxicillin, ampicillin, and levofloxacin [15, 18, 42, 43].

Our results showed that 86.9% of the *L. monocytogenes* isolates obtained from animals and humans were MDR, as bacterial isolates that exhibit resistance against three or more different antibiotic classes are considered MDR [44]; nearly the same percentages were reported in one other study [43], and a higher percentage (100%) was reported in another previous study [15]. This high prevalence of MDR in *L. monocytogenes* is alarming both in the human health and veterinary fields since it increases the difficulty of treating listeriosis [45].

Our data revealed that the mean MAR indices of *L. monocytogenes* isolated from animal and human samples were 0.435 and 0.375, respectively. These results are in agreement with those of a previous study in which the mean MAR index of *L. monocytogenes* isolated from animals was 0.47 [46] and another study in which the mean MAR index of *L. monocytogenes* isolated from humans was ˃ 0.2 [39]. Our results revealed that the mean MAR index of the animal isolates was greater than that of the human isolates, which confirmed the high resistance of *L. monocytogenes* of animal origin. All *L. monocytogenes* isolates in our study had an MAR of more than 0.2, which indicates that all the isolates originated from high-risk sources of contamination where antibiotics are often used [40].

Phylogenetic and sequence analyses based on the *inlB* gene revealed 100% identity between faecal, milk and stool *L. monocytogenes* isolates. Additionally, the strains shared 100% identity with many isolates from different sources, such as sheep placenta (accession no. URN79349), sheep brain (accession no. OM854788) and human origin (accession no. DQ302514). The high percentage of genetic similarity between isolates might imply that the isolates have not diverged much, as they shared a recent ancestor strain. This hypothesis would explain why the strains retain such similar *inlB* functionality. This result indicates that although these isolates are from different hosts, they have the ability to facilitate the invasion of host cells, which is key in the virulence of *L. monocytogenes*. These results suggests zoonotic transmission of *L. monocytogenes*, which is in agreement with the results reported by other authors and confirmed the importance of zoonotic *L. monocytogenes* transmission between animals and humans [40].

## Conclusion

This study revealed an alarming high prevalence of MDR *L. monocytogenes* in animal and human samples. Phylogenetic analysis of animal and human samples revealed 100% identity between human and animal isolates, which represent a great danger to public health due to the increasing possibility of zoonotic transmission of *L. monocytogenes* and treatment failure; therefore, restricting the use of antibiotics as prophylaxes and growth promoters on animal farms is recommended. Additionally, this disease can be prevented by improving farm hygiene and biosafety measures.

## Methods

### Ethical declaration

The collection of samples and study design were performed in accordance with the “Institutional Review Board” of the Faculty of Medicine at Assiut University. The Institutional Approval Number was 04-2023-200283. All farm owners included in this study were informed of all the study procedures and aims, and permission to collect animal samples was obtained from them verbally.

## Study area and design

**Fig. 5:**
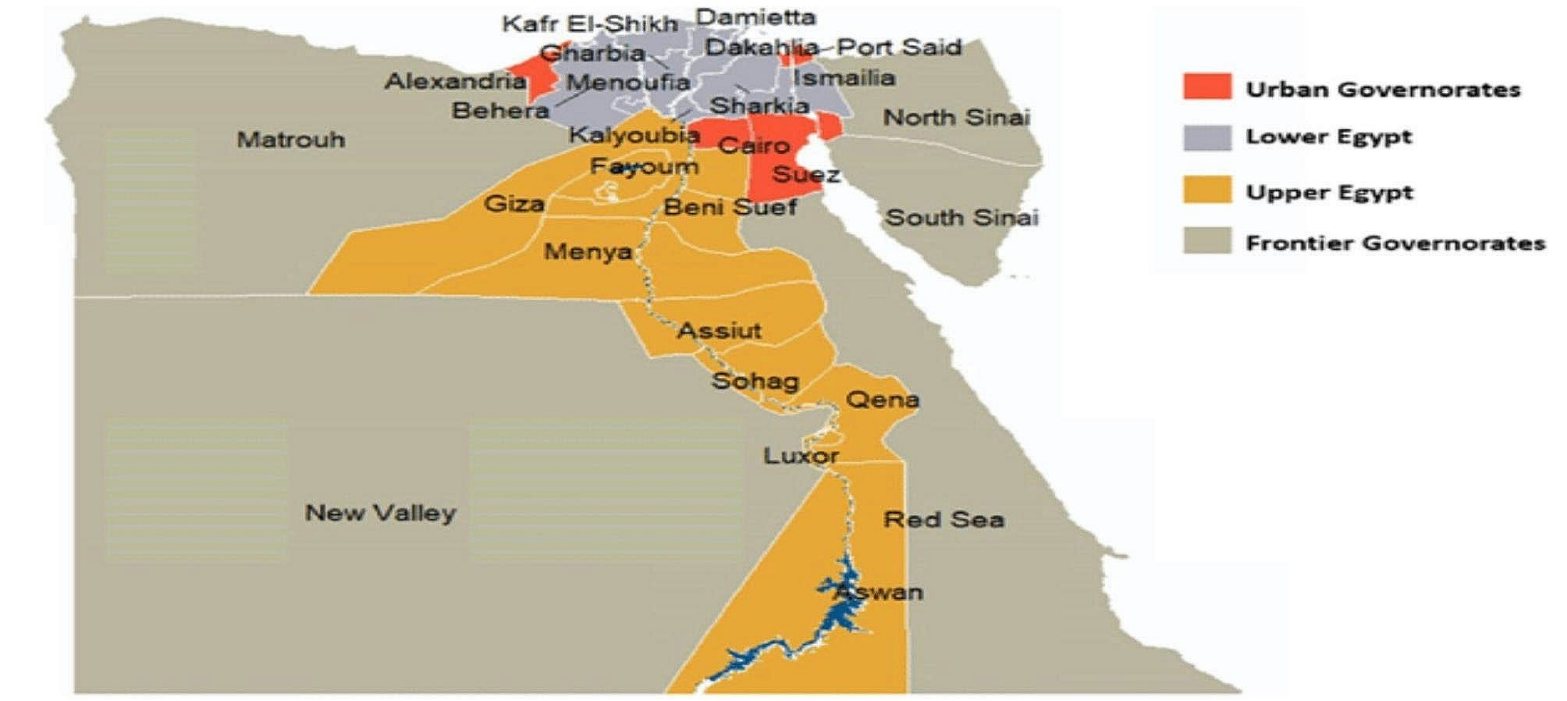
Map of Egypt Governorates (New Valley and Beheira Governorates)

Samples were collected from September 2022 to November 2023 in New Valley and Beheira Governorate, Egypt. The New Valley is the largest semiarid Governorate in Egypt, consisting of roughly half of Egypt’s area. This area is in the southwestern part of the country, between the Nile, southeastern Libya, and northern Sudan. This study included the main centre of the New Valley Governorate, El-Kharga. Beheira is a large coastal governorate that is located west of the Nile delta and is bounded by the Mediterranean Sea to the north and the Giza governorate to the south, while it is aligned with the Rosetta Nile branch in the east and the Alexandria and El-Alamein Governorates in the west, as shown in Fig. (5).

### Sampling

A total of 2097 samples were collected from different farm animals (cows, sheep, and goats) and humans. The samples included cow faeces (*n* = 541), sheep faeces (*n* = 319), goat faeces (*n* = 179), cow’s milk (*n* = 414), sheep milk (*n* = 173), and goat milk (*n* = 77), in addition to human stool samples (*n* = 394 from contacts and noncontact persons). These samples were collected according to methods described by Abdeen et al. (2021) and Wu et al. (2021) [33, 36].

### **Bacteriological examination of** *L. monocytogenes*

*L. monocytogenes* was isolated according to the instructions of the International Organization of Standards (ISO 11290-1) [47]. One gram of each sample was selectively enriched in nine ml of *Listeria* enrichment broth and incubated at 30 °C for 48 h. From each tube of *Listeria* enrichment broth culture, a loopful of sample was streaked onto Oxford agar plates and incubated at 37 °C for 48 h. The suspected colonies on Oxford agar were surrounded by a black halo. After purification of the suspected colonies, they were transferred onto tryptic soya agar with 6% yeast extract (TYASE), incubated at 37 °C for 24 h, and then maintained at 4 °C for biochemical identification.

### **Identification of** *L. monocytogenes*

#### Biochemical identification of *L. monocytogenes*

Biochemical identification was achieved using haemolysis tests, motility tests, catalase tests, and sugar fermentation tests according to methods described by Aygun and Pehlivanlar (2006) [48].

#### **Molecular identification of** *L. monocytogenes*

PCR was performed to confirm *L. monocytogenes* via the detection of *16 S rRNA* and to assess its pathogenicity via detection of the *inlB* virulence gene using the specific primers shown in Table (6) [49, 50]. Genomic DNA was extracted using a QIAamp DNA Mini Kit (catalogue no. 51,304). The Emerald Amp GT PCR master mix (Takara, Code No. RR310A kit) was used. The cycling protocol for the *L. monocytogenes 16 S rRNA* gene was as follows: 35 cycles of 94 °C for five min during primary denaturation, 94 °C for 30 s during secondary denaturation, 60 °C for 40 s during annealing and 72 °C for 1.2 min during extension, followed by a final extension at 72 °C for 12 min. Moreover, the PCR cycling protocol for *the inlB* gene was applied at 94 °C for five min during primary denaturation, 94 °C for 30 s during secondary denaturation, 55 °C for 40 s during annealing, and 72 °C for 40 s for 35 cycles during extension, followed by a final extension at 72 °C for ten min. Five microlitres of each amplicon was electrophoresed in a 1% agarose gel, stained with ethidium bromide and visualized and captured on a UV transilluminator. The marker for the PCR products was a 100 bp DNA ladder.

**Table 6:** Primer sequences for *L. monocytogenes 16 S rRNA* and *inlB* genes

Gene	Sequence	Amplified product	Reference
*16 S rRNA*	GGA CCG GGGCTAATACCGAATGATAA	1200 bp	[49]
	TTCATGTAGGCGAGTTGCAGCCTA		
*inl B*	CTGGAAAGTTTGTATTTGGGAAA	343 bp	[50]
	TTTCATAATCGCCATCATCACT		

### Phylogenetic analysis

A comparative sequencing analysis of one animal and one human *L. monocytogenes* isolate was performed using the *inlB* gene. The *inlB* gene sequences were analysed with the CLUSTRAL W multiple sequence alignment program, version 12.1 of the MegAlign module of Lasergene DNA Star Software Pairwise (Madison, Wisconsin, USA) [51]; phylogenetic analysis was performed using maximum parsimony in MEGA6 [52].

### **Antibiotic resistance profile of isolated** *L. monocytogenes*

The antibiotic sensitivity test was performed by the disc diffusion method according to the Clinical and Laboratory Standards Institute (CLSI) instructions [53]. The interpretation of inhibition zone diameters was carried out according to the clinical breakpoint value for *Staphylococcus* species, as no resistance standards exist in CLSI procedures for *L. monocytogenes* [54]. The efficacies of the antimicrobial discs used are shown in Table (7). The MAR index was calculated after dividing the number of antimicrobial agents to which the isolate was resistant by the total number of antimicrobial agents used [55, 56].

**Table 7:** Antimicrobial discs, concentration, and interpretation of their action on the isolated *L. monocytogenes*

Antimicrobial agent	Concentration (µg)	Symbol	sensitive (mm)	Resistant (mm)	Resistant (mm)
Amikacin	30	AK	≥ 17	15–16	≤ 14
Amoxcillin	10	AMX	≥ 17	14–16	≤ 13
Ampicillin	10	AMP	≥ 17	14–16	≤ 13
Apramycin	15	APR	≥ 17	15–16	≤ 15
Flumequine	36	FLM	≥ 18	13–17	≤ 12
Gentamicin	10	CN	≥ 15	13–14	≤ 12
Levofloxacin	5	LEV	≥ 19	16–18	≤ 15
Lincomycin	10	MY	≥ 21	---	≤ 20
Norfloxacin	10	NOR	≥ 17	13–16	≤ 12
Rifampicin	5	RD	≥ 20	17–19	≤ 16
Trimethoprime – sulfamethoxazole	1.25-23	SXT	≥ 16	11–15	≤ 10

### Statistical analysis

The data were statistically analysed using the chi-square test in SPSS ver. 27 (IBM Corp. Released 2013) to predict associations between variables. The data were treated as a complete randomization design according to methods described by Steel et al. (1997) [57]. P values < 0.05 were considered significant. The significance level was set at < 0.05.

### Electronic supplementary material

Below is the link to the electronic supplementary material.

Supplementary Material 1

## Data Availability

The datasets used and/or analyzed in the current study were not publicly published to preserve the privacy of the participants but are available upon reasonable request from the corresponding author.
