# Supplementary material for: Prevalence, antibiogram and molecular characterization of Listeria monocytogenes from ruminants and humans in New Valley and Beheira Governorates, Egypt
Source: BMC Vet Res. 2024 Jul 6;20:297. doi: 10.1186/s12917-024-04138-0 (PMC11227151; doi:10.1186/s12917-024-04138-0)
Supplement: Supplementary file 1 — Supplementary Material 1 [file 12917_2024_4138_MOESM1_ESM.docx]

**Original unprocessed photo**


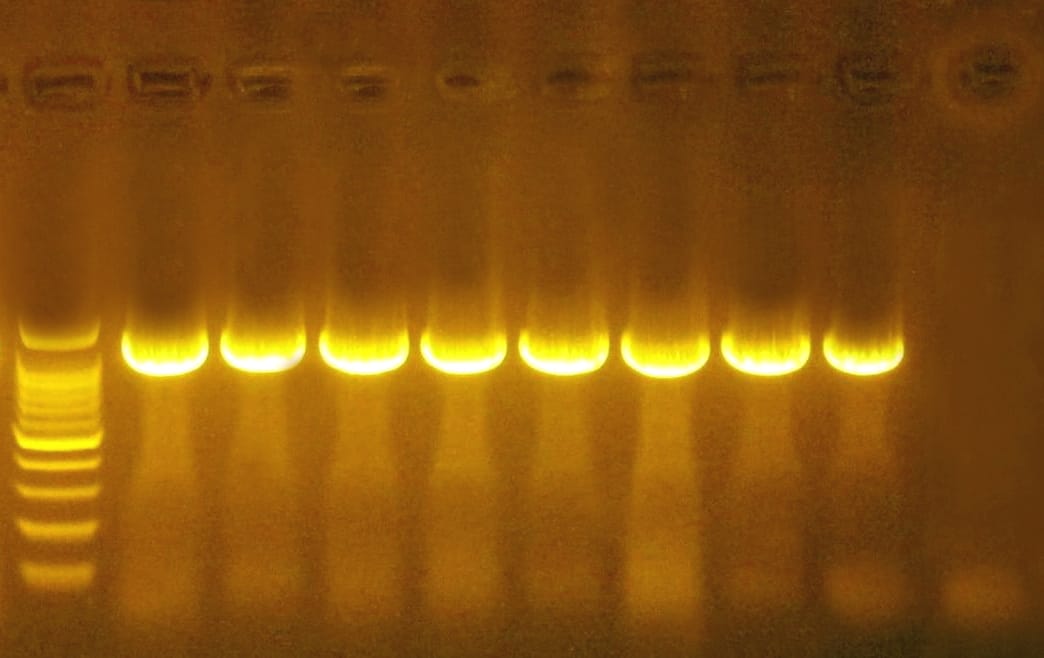


**Figure (1)**: Agarose gel electrophoresis of amplified *16s rRNA* gene PCR product (1200 bp).


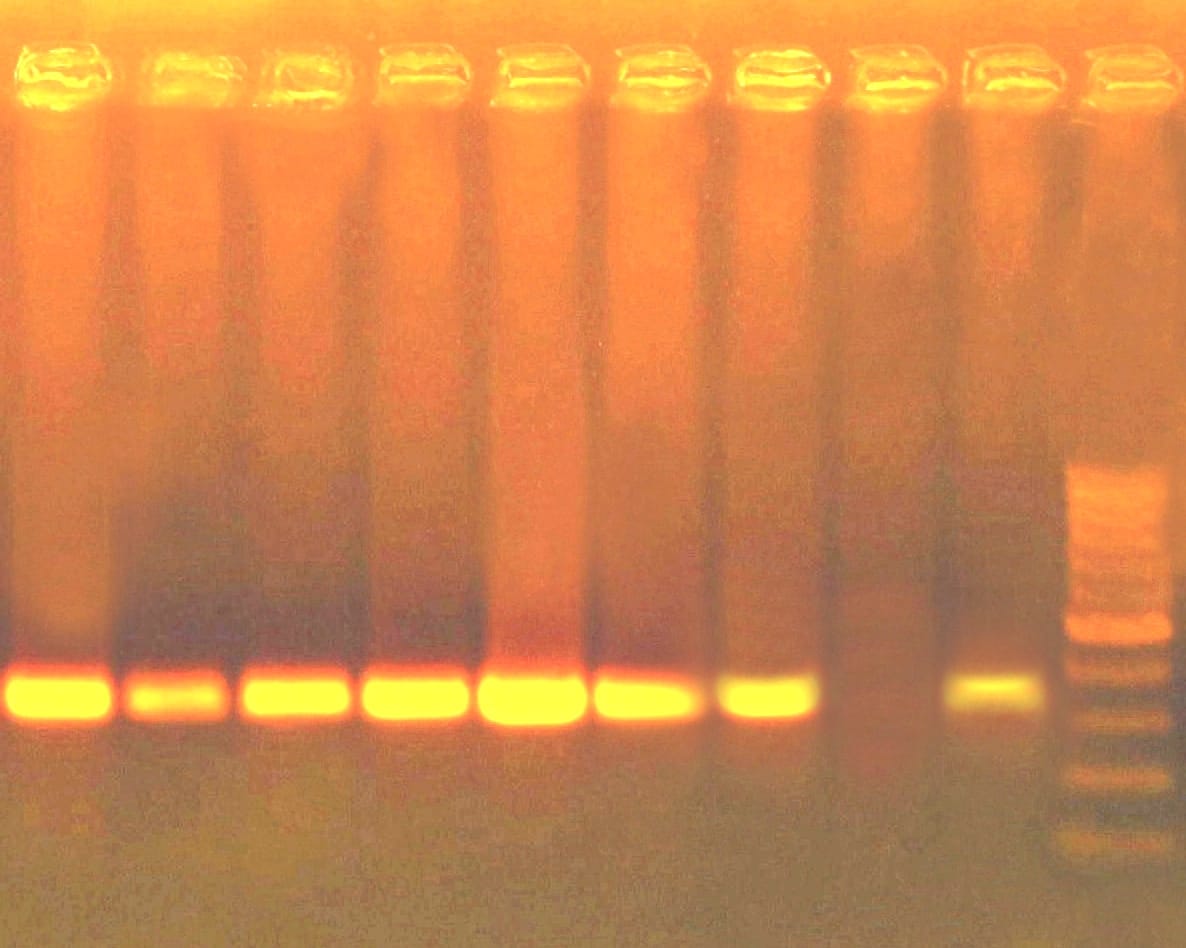


**Figure (2):** Agarose gel electrophoresis of amplified *inlB* gene PCR product (343 bp).
